# Contagem de linfócitos e concentrações séricas de albumina e transferrina em pacientes submetidos à artroplastia total de joelho

**DOI:** 10.1055/s-0045-1809530

**Published:** 2025-07-25

**Authors:** Bernardo Crespo Alves, Amanda S. Cavalcanti, Kelly Biancardini Gomes Barbato, João Maurício Barretto, Juliana Arruda de Matos

**Affiliations:** 1 Centro de Atenção Especializada ao Joelho, Instituto Nacional de Traumatologia e Ortopedia, Rio de Janeiro, RJ, Brasil; 2 Hospital Clementino Fraga Filho, Universidade Federal do Rio de Janeiro (UFRJ), Rio de Janeiro, Brasil; 3 Divisão de Pesquisa, Instituto Nacional de Traumatologia e Ortopedia, Rio de Janeiro, RJ, Brasil; 4 Grupo de Joelho do Hospital São Vicente, Rede D'Or, Rio de Janeiro, RJ, Brasil; 5 Comissão de Controle de Infecção Hospitalar, Instituto Nacional de Traumatologia e Ortopedia, Rio de Janeiro, RJ, Brasil

**Keywords:** joelho, osteoartrite, artroplastia do joelho, desnutrição, infecção articular periprotética, knee, osteoarthritis, arthroplasty, replacement, knee, malnutrition, periprosthetic joint infection

## Abstract

**Objetivo:**

Descrever a prevalência de desnutrição pré-operatória em indivíduos submetidos à ATJ primária e avaliar sua associação à idade, sexo, índice de massa corporal (IMC) e comorbidades, bem como o risco de hospitalização pós-operatória prolongada, infecção articular periprotética (IAP) precoce ou readmissão.

**Métodos:**

Este é um estudo de coorte de ATJs realizadas entre 2014 e 2016. A desnutrição pré-operatória foi definida pelo número total de linfócitos < 1.500 células/mm ^3^ , concentração sérica de albumina < 3,5 g/dL ou concentração de transferrina < 200 mg/dL nos 6 meses anteriores à cirurgia.

**Resultados:**

Das 2.080 ATJ realizadas, 1.099 casos tinham dados válidos de linfometria, albumina e transferrina e foram incluídas na análise. A prevalência de desnutrição foi de 17,7%. Os fatores independentes associados à maior prevalência de desnutrição foram idade (razão de chances [OR] = 1,03; intervalo de confiança [IC] de 95% = 1,01-1,05), anemia (1,55 [1,05-2,28]), baixo peso (3,13 [1,50-6,50]) e peso normal (1,85 [1,21-2,82]). O diabetes mellitus foi inversamente associado à desnutrição (0,60 [0,38-0,96]). A IAP precoce foi diagnosticada em 18 (1,6%) participantes. Não houve associação estatisticamente significativa e independente entre desnutrição e complicações pós-cirúrgicas.

**Conclusão:**

As alterações em números de linfócitos e concentrações séricas de albumina e transferrina são comuns em indivíduos submetidos à ATJ, em especial em pacientes mais velhos, anêmicos e com peso normal ou baixo. Estudos futuros com amostras maiores são necessários para avaliar melhor a relação entre desnutrição e desfechos adversos após ATJ.

## Abstract (en)

**Objective:**

To describe the prevalence of preoperative malnutrition in individuals undergoing primary TKA and to assess its association with age, sex, body mass index (BMI), and comorbidities, as well as the risk of prolonged postsurgical hospitalization, early prosthetic joint infection (PJI), or readmission.

**Methods:**

We conducted a cohort study of TKAs performed between 2014 and 2016. Preoperative malnutrition was defined as a total lymphocyte count < 1,500 cells/mm ^3^ , a serum albumin concentration < 3.5 g/dL, or a transferrin concentration < 200 mg/dL within the six months before surgery.

**Results:**

Out of the 2080 TKAs performed, 1099 had valid lymphometry, albumin, and transferrin data and were included in the analysis. The prevalence of malnutrition was 17.7%. Independent factors associated with a higher prevalence of malnutrition were age (OR = 1.03; 95% CI = (1.01–1.05)), anemia (1.55 (1.05–2.28)), low weight (3.13 (1.50–6.50)), and normal weight (1.85 (1.21–2.82)). Diabetes mellitus was inversely associated with malnutrition (0.60 (0.38–0.96)). Early PJI was diagnosed in 18 (1.6%) participants. There was no statistically significant and independent association between malnutrition and postsurgical complications.

**Conclusion:**

Altered lymphocyte count, serum albumin, and transferrin levels is common among individuals undergoing TKA, particularly in older patients, those with anemia, and individuals with normal or low weight. Future studies with larger sample sizes are needed to better assess the relationship between malnutrition and adverse outcomes following TKA.

## Introdução

A desnutrição é um desequilíbrio na ingestão calórica, proteica ou vitamínica que leva à depleção nutricional aguda ou crônica e comprometimentos metabólicos ou funcionais. 1 Essa desnutrição pode ser decorrente da redução da ingesta alimentar, distúrbios de absorção, estados hipercatabólicos ou condições de consumo. 2 Diversos critérios diagnósticos, incluindo índices antropométricos (por exemplo, índice de massa corporal e análise da composição corporal), questionários nutricionais e marcadores séricos, são usados para definir a desnutrição, cada um com suas limitações e vantagens.

Apesar da disponibilidade de vários métodos para triagem e avaliação da desnutrição, como medidas antropométricas, análise da composição corporal, questionários nutricionais e marcadores laboratoriais, o diagnóstico da desnutrição ainda é impreciso. A Organização Mundial da Saúde (OMS) define a desnutrição como índice de massa corporal (IMC) abaixo de 18,5 kg/m ^2^ . Em contrapartida, a *American Society for Parenteral and Enteral Nutrition* (ASPEN) a define com base na presença de dois ou mais critérios, incluindo ingestão calórica inadequada, perda de peso, massa muscular ou gordura subcutânea, acúmulo localizado ou generalizado de fluidos que pode mascarar a perda de peso e diminuição da capacidade funcional. 2 A *European Society for Clinical Nutrition and Metabolism* (ESPEN) propôs um consenso para o diagnóstico de desnutrição que combina o uso de questionários de risco nutricional e critérios como IMC, perda relativa de peso ou índice de massa magra. 3 Este consenso tem sido criticado pela sua impraticabilidade, falta de orientações claras sobre o questionário de risco nutricional a ser empregado e não consideração de alterações bioquímicas inflamatórias envolvidas no processo de desnutrição. 4 5 Os marcadores séricos são bastante utilizados para avaliação nutricional devido à sua acessibilidade, reprodutibilidade e capacidade de detectar alterações agudas no estado nutricional. Parâmetros comuns para identificação de desnutrição são o número total de linfócitos abaixo de 1.500 células/mm ^2^ , as concentrações séricas de albumina abaixo de 3,5 g/dL e as concentrações de transferrina abaixo de 200 mg/dL. 6 Outros marcadores nutricionais, como pré-albumina, interleucinas, proteína C reativa e leptina, são utilizados com menor frequência. 7

A associação entre a desnutrição e as complicações pós-operatórias foi observada pela primeira vez em cirurgias abdominais. 8 Na ortopedia, a desnutrição tem sido associada a desfechos piores em pacientes com fraturas proximais do fêmur, aumentando o tempo das internações hospitalares, as complicações infecciosas e não infecciosas e as taxas de readmissão. 9 10

A artroplastia total de joelho (ATJ) é uma cirurgia complexa que visa tratar a osteoartrite avançada do joelho, restaurando a função e aliviando os sintomas. As projeções indicam uma demanda crescente por artroplastias, com impacto significativo nos custos do sistema de saúde público e privado. 11 A infecção articular periprotética (IAP) é a principal causa de falha precoce e cirurgia de revisão em curto prazo. 12 Pesquisas sobre a relação entre desnutrição e IAP, bem como complicações não infecciosas em artroplastias, vêm ganhando destaque na literatura. 13 14 Entretanto, os dados brasileiros são escassos, o que ressalta a necessidade de avaliar o estado nutricional para compreender sua influência e desenvolver estratégias de mitigação de riscos.

O objetivo deste estudo foi avaliar a prevalência de desnutrição pré-operatória e sua associação a características demográficas e clínicas, bem como o risco de IAP e complicações não infecciosas pós-ATJ em um centro de referência em cirurgia ortopédica.

## Materiais e Métodos

Este estudo de coorte é uma análise retrospectiva de dados coletados prospectivamente e foi aprovado pelo Comitê de Ética em Pesquisa (CAAE 63639216.3.0000.5273). Foram incluídos todos os pacientes submetidos à ATJ primária entre janeiro de 2014 e dezembro de 2016. Foram excluídos pacientes com artroplastias bilaterais simultâneas ou não residentes no Estado do Rio de Janeiro.

Todas as cirurgias foram realizadas por cirurgiões especialistas em joelho. Os pacientes receberam profilaxia antimicrobiana 30 a 60 minutos antes da incisão e foram transferidos para a unidade de terapia intensiva no primeiro dia pós-operatório. A alta hospitalar ocorreu a partir do terceiro dia de internação sob condições clínicas adequadas e controle da dor. A prevenção de eventos tromboembólicos foi realizada com a administração de uma dose única diária subcutânea de 40 mg de heparina de baixo peso molecular (Clexane®, Sanofi Aventis), iniciada 12 a 24 horas após a cirurgia e mantida por 14 dias. O acompanhamento pós-operatório dos pacientes foi realizado por meio de consultas ambulatoriais regulares.

O diagnóstico de IAP profunda seguiu as diretrizes da *Musculoskeletal Infection Society* (MSIS). 15 A classificação temporal da IAP foi baseada no intervalo entre o procedimento-índice e o início dos sintomas: precoce (< 3 meses), intermediária (3 meses a 2 anos) ou tardia (> 2 anos). 16 Analisamos apenas infecções precoces devido à sua associação direta com fatores de risco perioperatórios.

A desnutrição foi definida por pelo menos um dos seguintes parâmetros laboratoriais: número total de linfócitos < 1.500 células/mm ^3^ e concentração sérica de albumina < 3,5 g/dL ou transferrina < 200 mg/dL. 6 Nos pacientes com mais de 60 anos, o IMC foi categorizado de acordo com as recomendações da Organização Pan-Americana da Saúde (OPAS): abaixo do peso (< 23 kg/m ^2^ ), normal (23 a 27,9 kg/m ^2^ ), sobrepeso (28 a 29,9 kg/m ^2^ ) e obeso (> 30 kg/m ^2^ ). 17 Nos indivíduos com menos de 60 anos, o IMC foi categorizado usando as classificações modificadas da OMS: abaixo do peso (< 18,5 kg/m ^2^ ), normal (18,5 a 24,9 kg/m ^2^ ), sobrepeso (25 a 29,9 kg/m ^2^ ) ou obesidade (> 30 kg/m ^2^ ). A anemia foi definida pelos critérios da OMS com base no sexo, com concentração de hemoglobina <12 g/dL em mulheres e <13 g/dL em homens. 18

O banco de dados do hospital foi pesquisado para obter informações sobre todos os pacientes submetidos à ATJ primária entre 2014 e 2016. Os dados coletados incluíram idade, sexo ao nascer, registros médicos, peso, altura, comorbidades (como hipertensão, diabetes, asma, cardiopatia, dislipidemia e artrite reumatoide), classificação da *American Society of Anesthesiologists* (ASA), data da cirurgia, datas de admissão e alta, motivo da alta, transfusão de sangue perioperatória e resultados laboratoriais pré-operatórios (hemoglobina, hematócrito, albumina, transferrina e linfócitos). Os exames laboratoriais válidos foram aqueles realizados até 180 dias antes da cirurgia; na presença de múltiplos resultados, o mais próximo da data da cirurgia foi usado para análise. Novas admissões em até 90 dias após o procedimento foram revistas em prontuários médicos físicos. Além disso, registros de vigilância epidemiológica do Comitê de Controle de Infecção Hospitalar foram utilizados para identificar pacientes com IAP precoce. Os dados de ambas as fontes foram inseridos em uma planilha do Microsoft Excel 2016®.

As variáveis quantitativas foram apresentadas como medianas e intervalos interquartis (IIQs) e valores mínimos e máximos, enquanto as variáveis categóricas foram expressas como frequência (n) e porcentagem (%). O teste de Shapiro-Wilk avaliou a normalidade da amostra. O teste t de Student paramétrico foi usado em dados com distribuição normal e ajustes para variâncias desiguais conforme necessário. Os testes de Mann-Whitney ou Kruskal-Wallis foram empregados em dados não normais. As variáveis categóricas foram analisadas usando os testes qui-quadrado ou exato de Fisher, como apropriado. As razões de chances (OR) avaliaram a associação entre desnutrição e as variáveis explicativas. A regressão de risco proporcional analisou o tempo de hospitalização pós-operatória e tratou o tempo como uma variável distinta devido à sua distribuição não normal (comando *pgmhaz* ). A regressão logística identificou fatores independentes associados à desnutrição. O teste de McNemar comparou a prevalência de desnutrição com base em diferentes critérios. A significância estatística foi estabelecida em 5% ( *p* < 0,05) e os dados foram analisados em Stata® versão 17.0 (StataCorp LLC, College Station, Texas, EUA).

## Resultados

**Fig. 1:**
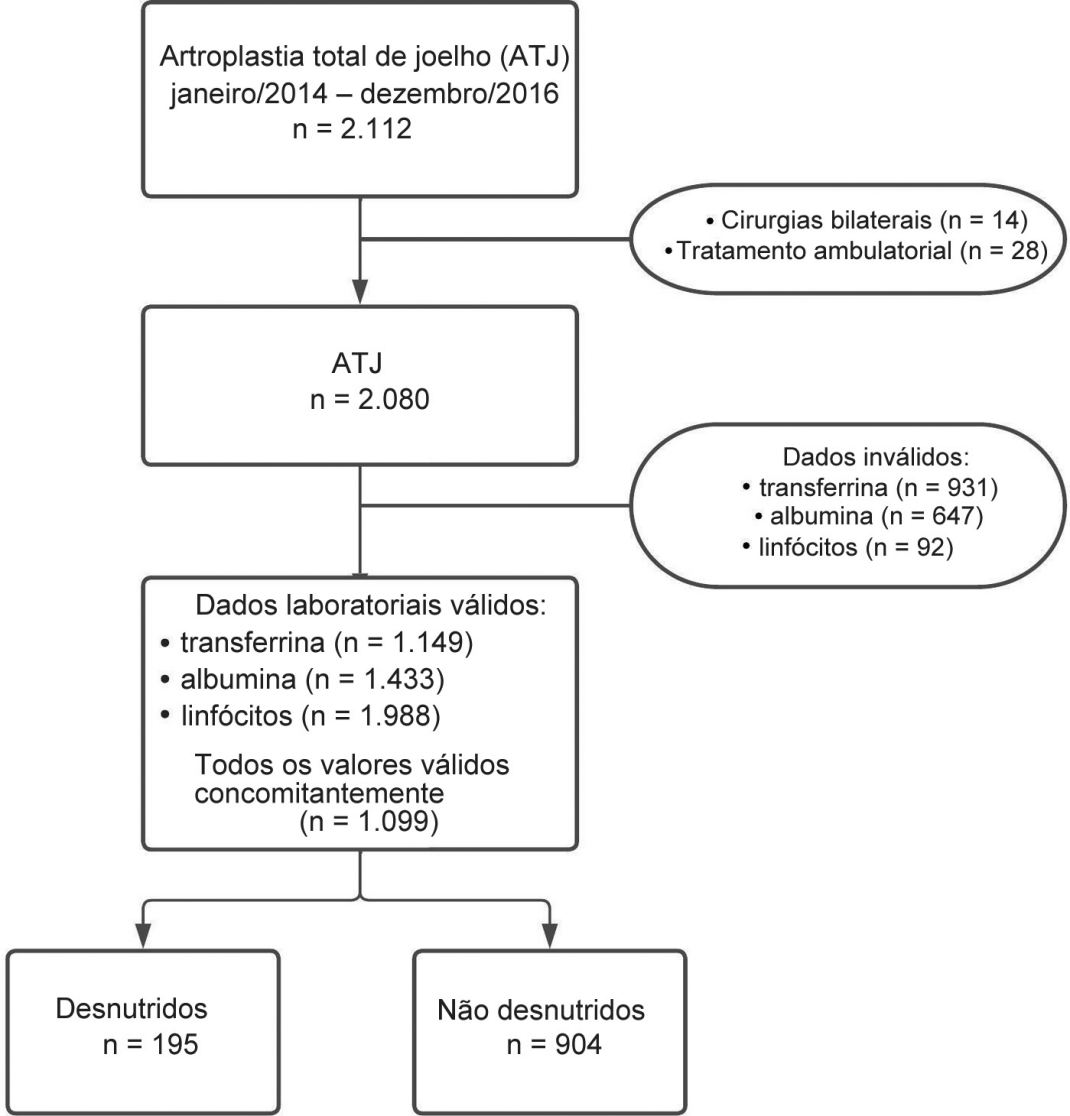
Fluxograma de inclusão de participantes no estudo.

Entre 1° de janeiro de 2014 e 31 de dezembro de 2016, foram realizadas 2.112 ATJs primárias. Após a aplicação dos critérios de exclusão (14 ATJs bilaterais simultâneas e 28 pacientes de fora do estado do Rio de Janeiro), foram analisadas 2.080 ATJs ( Fig. 1 ). Destas, 73,6% ocorreram em pacientes do sexo feminino, com idade mediana de 68 anos (IIQ, 63-74). A Tabela Suplementar S1 mostra os dados completos dos pacientes.

**Fig. 2:**
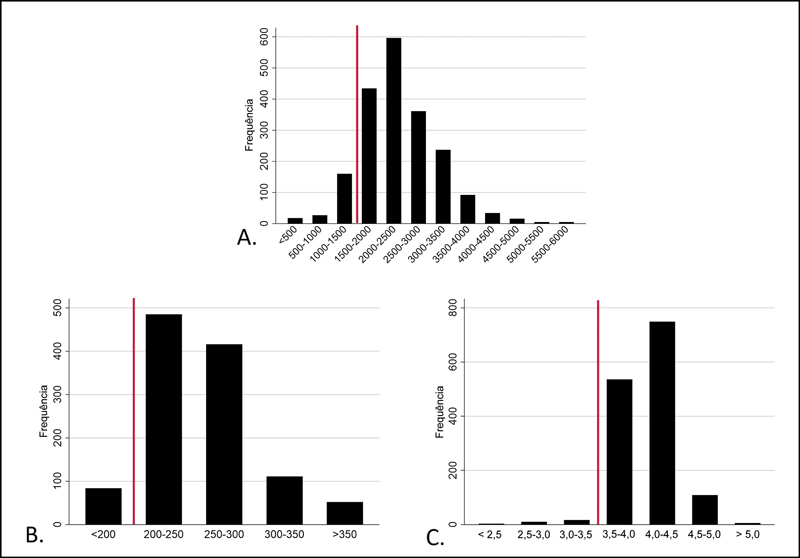
Distribuição dos marcadores laboratoriais de desnutrição, medidos até 180 dias antes da artroplastia total primária de joelho. A linha vermelha em cada gráfico indica o ponto de corte para o critério de desnutrição. 3 A. Contagem de linfócitos ( *n* = 1.988): 205 participantes com valores abaixo de 1.500 células/mm ^3^ ; B. Concentração de transferrina ( *n* = 1.149): 84 participantes com valores abaixo de 200 mg/dL; C. Concentração de albumina ( *n* = 1.433): 30 participantes com valores abaixo de 3,5 g/dL.

Resultados válidos de albumina sérica, transferrina total e número total de linfócitos foram obtidos de 1.433, 1.149 e 1.988 participantes, respectivamente. A concentração sérica mediana de albumina foi de 4,0 mg/dL (IIQ 3,8-4,2); 30 pacientes (2,1%) apresentaram hipoalbuminemia (< 3,5 g/dL). A concentração mediana de transferrina foi de 250 mg/dL (IIQ, 226-279 mg/dL) e 84 pacientes (7,3%) apresentaram valores abaixo de 200 mg/dL. O número total mediano de linfócitos foi de 2.288 células/mm ^2^ (IIQ, 1.870-2.834) e 205 pacientes (10,3%) apresentaram menos de 1.500 células/mm ^3^ ( Fig. 2 e Tabela Suplementar S2 ).

**Fig. 3:**
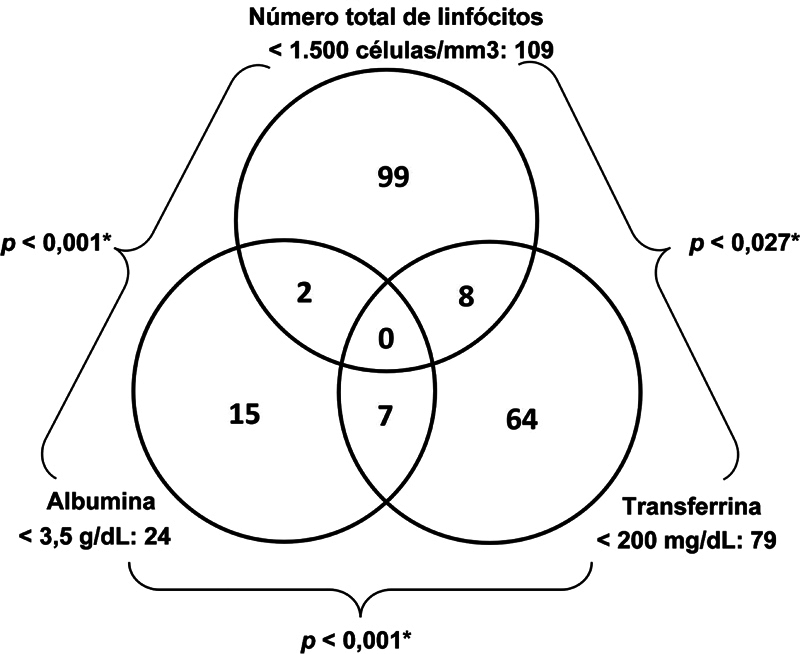
Distribuição dos 195 pacientes classificados como desnutridos (dentre os 1.099 com os três parâmetros laboratoriais disponíveis e válidos) segundo a desnutrição definida laboratorialmente.

Dos 2.080 participantes, 1.099 apresentaram dados válidos de albumina, transferrina e linfócitos; 195 foram categorizados como desnutridos (17,7%) e 904 como não desnutridos (82,3%). Entre os desnutridos, 17 indivíduos (8,7%) apresentaram anomalias concomitantes em dois ou mais marcadores séricos. A análise de concordância pareada revelou uma diferença estatisticamente significativa ( *p* < 0,05), indicando baixa sobreposição entre esses marcadores ( Fig. 3 ).

Os indivíduos desnutridos tinham mais idade e apresentavam hematócrito, número de leucócitos e IMC menores do que seus pares não desnutridos. A prevalência de homens foi maior entre os desnutridos ( *p* > 0,05). Os pacientes desnutridos também apresentaram maiores taxas de classificação de baixo peso e peso normal, maior prevalência de anemia e transfusões de sangue em comparação aos não desnutridos. Hipertensão e diabetes foram mais comuns entre indivíduos sem desnutrição definida por valores laboratoriais ( Tabela 1 ).

**Tabela 1:** Análise das características demográficas e laboratoriais de pacientes desnutridos e não desnutridos submetidos à artroplastia total primária de joelho ( *n* = 1.099)

	Não desnutrido ( *n* = 904)		Desnutrido ( *n* = 195)		Total		OR	IC de 95%	Valor de p
Sexo – N (%)									
Feminino	678	(75,0%)	133	(68,2%)	811	(73,8%)			
Masculino	226	(25,0%)	62	(31,8%)	288	(26,2%)	1,40	(1,00, 1,96)	0,051
IMC (OMS/OPAS) – N (%)									
Obesidade	480	(57,7%)	73	(42,2%)	553	(55,0%)	1,00	–	–
Sobrepeso	139	(16,7%)	33	(19,1%)	172	(17,1%)	1,56	(0,99, 2,45)	0,054
Peso normal	188	(22,6%)	53	(30,6%)	241	(24,0%)	1,85	(1,25, 2,74)	**0,002**
Subpeso	25	(3,0%)	14	(8,1%)	39	(3,9%)	3,65	(1,83, 7,41)	**< 0,001**
ASA – N (%)									
I	143	(21,3%)	30	(21,3%)	173	(21,3%)	1,00	–	–
II	523	(77,9%)	110	(78,0%)	633	(78,0%)	1,00	(0,64, 1,56)	0,991
III	5	(0,7%)	1	(0,7%)	6	(0,7%)	0,95	(0,11, 8,46)	0,966
Hipertensão arterial – N (%)									
Não	219	(24,3%)	61	(31,3%)	280	(25,5%)			
Sim	683	(75,7%)	134	(68,7%)	817	(74,5%)	0,70	(0,50, 0,99)	**0,043**
Diabetes mellitus – N (%)									
Não	669	(74,0%)	167	(85,6%)	836	(76,1%)			
Sim	235	(26,0%)	28	(14,4%)	263	(23,9%)	0,48	(0,31, 0,73)	**0,001**
Cardiopatia – N (%)									
Não	852	(94,2%)	185	(94,9%)	1037	(94,4%)			
Sim	52	(5,8%)	10	(5,1%)	62	(5,6%)	0,89	(0,44, 1,77)	0,732
Dislipidemia – N (%)									
Não	853	(94,4%)	187	(95,9%)	1040	(94,6%)			
Sim	51	(5,6%)	8	(4,1%)	59	(5,4%)	0,72	(0,33, 1,53)	0,389
Artrite reumatoide – N (%)									
Não	858	(94,9%)	180	(92,8%)	1038	(94,5%)			
Sim	46	(5,1%)	14	(7,2%)	60	(5,5%)	1,45	(0,78, 2,70)	0,239
Anemia – N (%)									
Não	632	(77,4%)	118	(68,2%)	750	(75,8%)			
Sim	185	(22,6%)	55	(31,8%)	240	(24,2%)	1,59	(1,11, 2,28)	**0,011**
Transfusão de sangue – N (%)									
Não	777	(86,6%)	154	(79,0%)	931	(84,7%)			
Sim	127	(14,1%)	41	(21,0%)	168	(15,3%)	1,63	(1,10, 2,41)	0,015
Idade (anos) – mediana (IIQ)	67	(63–73)	70	(64–76)	68	(63–74)	–	–	0,001
Hematócrito (%) – mediana (IIQ)	39,0	(36,6–41,9)	38,2	(35,6–41,1)	38,9	(36,4–41,7)	–	–	0,012
Hemoglobina (g/dL) – mediana (IIQ)	12,9	(12,1–13,9)	12,9	(11,9–13,8)	12,9	(12,0–13,9)	–	–	0,113
Leucócitos (células/mm ^3^ ) – mediana (IIQ)	7300	(6300–8600)	5900	(4900–7700)	7200	(6000–8500)	–	–	< 0,001
IMC (kg/m ^2^ ) – mediana (IIQ)	30,8	(27,5–34,7)	29,2	(26,0–32,7)	30,6	(27,3–34,5)	–	–	< 0,001
Volume de transfusão de sangue (mL) – mediana (IIQ)	290	(256–513)	288	(261–511)	290	(257–512)	–	–	0,973

Abreviaturas: ASA, Pontuação de risco da American Society of Anesthesiologists; IC, Intervalo de confiança; IIQ, intervalo interquartil; IMC, índice de massa corporal; OMS, Organização Mundial de Saúde; OPAS, Organização Pan-Americana de Saúde; OR, razão de chances.

A idade, o baixo peso, o peso normal e a anemia foram fatores independentes associados a uma maior prevalência de desnutrição, enquanto o diabetes mellitus apresentou associação inversa ( Tabela 2 ). Esta análise incluiu 901 participantes com dados completos de todas as variáveis.

**Tabela 2:** Resultado do modelo de regressão logística multivariada dos fatores associados à desnutrição em pacientes ( *n* = 901) submetidos à artroplastia total primária de joelho

	OR	IC de 95%	Valor de p
Idade (anos)	1,03	(1,01, 1,05)	0,014
IMC (OMS/OPAS)			
Obesidade	1,00	–	–
Sobrepeso	1,61	(0,99, 2,62)	0,057
Peso normal	1,85	(1,21, 2,82)	0,005
Subpeso	3,13	(1,50, 6,50)	0,002
Diabetes mellitus	0,60	(0,38, 0,96)	0,031
Anemia	1,55	(1,05, 2,28)	0,028
*Intercepto*	*0,02*	*(0,00, 0,10)*	*0,000*

Abreviaturas: IC, Intervalo de confiança; IIQ, intervalo interquartil; IMC, índice de massa corporal; OMS, Organização Mundial de Saúde; OPAS, Organização Pan-Americana de Saúde; OR, razão de chances.

À avaliação de desfechos desfavoráveis após a ATJ primária, a desnutrição ( *p* = 0,025) e a concentração de transferrina ( *p* = 0,021) foram associadas ao tempo de hospitalização pós-operatória na análise bivariada. No entanto, outros desfechos, como IAP precoce, readmissão por qualquer causa e readmissão devido a complicações da ferida operatória, não foram correlacionadas ao estado nutricional ou marcadores séricos ( Tabela 3 ). Na análise multivariada, apenas a anemia apresentou associação independente à duração da internação pós-operatória ( Tabela 4 ). A Tabela Suplementar S3 apresenta a análise bivariada dos fatores relacionados ao tempo de internação.

**Tabela 3:** Análise da desnutrição e marcadores sorológicos nutricionais como fator de risco para desfechos desfavoráveis após a artroplastia total primária de joelho

	infecção articular periprotética precoce				Readmissão hospitalar				Readmissão hospitalar por complicações da ferida				Dias entre a cirurgia e a alta hospitalar	
		OR	IC de 95%	p		OR	IC de 95%	p		OR	IC de 95%	p	Mediana (IIQ)	p
Desnutridos ( *n* = 195)	3 (1,5%)	0,93	0,17- 3,32	0,904	17 (8,7%)	0,91	0,49- 1,59	0,730	8 (4,1%)	0,70	0,28- 1,52	0,358	4 (3–5)	**0.025**
Não desnutridos ( *n* = 904)	15 (1,7%)				86 (9,5%)				52 (5,8%)				3 (3–5)	
Albumina < 3,5 g/dL ( *n* = 30)	1 (3,3%)	2,16	0,51–14,35	0,447	3 (10,0%)	1,15	0,22–3,81	0,825	2 (6,7%)	1,26	0,14–5,18	0,751	4 (3–6)	0.149
Albumina > 3,5 g/dL ( *n* = 1403)	22 (1,6%)				124 (8,8%)				75 (5,4%)				3 (3–5)	
Transferrina < 200 mg/dL ( *n* = 84)	1 (1,2%)	0,66	0,02–4,28	0,689	5 (6,0%)	0,62	0,19–1,55	0,304	4 (4,8%)	0,88	0,23–2,48	0,816	4 (3–6)	**0.021**
Transferrina > 200 mg/dL ( *n* = 1065)	19 (1,8%)				99 (9,3%)				57 (5,4%)				3 (3–5)	
Linfócitos < 1.500 células/mm ^3^ ( *n* = 205)	6 (2,9%)	1,51	0,51–3,68	0,358	22 (10,7%)	1,19	0,71–1,93	0,458	12 (5,9%)	1,1	0,54–2,07	0,752	4 (3–5)	0.055
Linfócitos > 1.500 células/mm ^3^ ( *n* = 1783)	35 (2,0%)				163 (9,1%)				95 (5,3%)				3 (3–5)	

Abreviaturas: IC, Intervalo de confiança; IIQ, intervalo interquartil; IMC, índice de massa corporal; OR, razão de chances.

**Tabela 4:** Análise multivariada de fatores associados à artroplastia total primária de joelho e intervalo de alta hospitalar

	Razão de risco	IC de 95%	Valor de p
Idade (anos)	0,995	(0,980, 1,000)	0,056
Anemia	0,843	(0,754, 0,941)	**0,002**
*Intercepto*	*0,352*	*(0,248–0,499)*	*< 0,001*

Abreviaturas: IC, Intervalo de confiança.

## Discussão

Neste estudo, observamos uma prevalência de desnutrição definida laboratorialmente de 17,7% entre 1.099 pacientes submetidos à ATJ primária. A desnutrição foi associada à idade avançada, peso baixo e normal e anemia, mas inversamente relacionada ao diabetes. Não houve associação independente entre a desnutrição e a IAP ou complicações não infecciosas após a ATJ.

A prevalência de hipoalbuminemia foi menor do que a relatada na literatura e apenas 2,1% dos pacientes atenderam ao critério de desnutrição. Nelson et al. (2015) 19 observaram que 4,2% dos mais de 37.000 pacientes submetidos à ATJ primária nos Estados Unidos apresentavam níveis séricos baixos de albumina. Na Coreia do Sul, Morey et al. (2016) 20 relataram 7,1% de desnutrição com base nos níveis de albumina. Essa variação pode ser explicada por diferenças geográficas nos perfis alimentares, já que dados globais mostram maior ingestão de proteína total e animal no Brasil em comparação aos Estados Unidos e à Coreia do Sul. 21

Utilizando os níveis séricos de transferrina, a desnutrição foi identificada em 7,3% da amostra. Huang et al. (2013) 22 observaram 6,6% de desnutrição em pacientes submetidos à artroplastia eletiva. Embora o estudo tenha associado a desnutrição a maiores taxas de complicações, nossos resultados foram diferentes. Os autores também relataram baixa sobreposição entre albumina e transferrina, com 4,9% dos pacientes desnutridos apresentando alterações nos dois marcadores, semelhante aos 3,6% do nosso estudo.

O número total de linfócitos foi o marcador de desnutrição mais comum em nossa coorte (10,3%). Este achado condiz com estudos anteriores, como o de Morey et al. (2016), 20 que encontraram uma incidência de 16%. A sobreposição entre os marcadores de linfócitos e albumina foi rara, ocorrendo em apenas 1% dos casos em nosso estudo e 7,7% em Morey et al. (2016). 20 A ausência de sobreposição entre os marcadores nutricionais é notável, visto que a desnutrição tipicamente afeta a síntese proteica e a imunidade; no entanto, alterações concomitantes em albumina, transferrina e linfócitos não foram notadas aqui ou em estudos anteriores. 20 22

Nosso estudo observou uma associação pequena, mas estatisticamente significativa, entre desnutrição e idade avançada, provavelmente decorrente de fatores como redução do apetite e da ingesta alimentar, comorbidades, polifarmácia e declínio funcional. 23 O isolamento familiar também é importante, uma vez que o aumento da dependência, a perda de autonomia e a falta de apoio doméstico têm impacto negativo na nutrição. 24 Esses problemas eram comuns em nossa população, muitas vezes devido à perda de mobilidade por doenças ortopédicas.

A anemia indica mau estado nutricional, especialmente em idosos. 25 Em nosso estudo, os níveis de transferrina foram usados para avaliar a desnutrição, mas variam de acordo com o tipo de anemia: aumentam na deficiência de ferro para otimizar a absorção do íon e diminuem na doença crônica, juntamente com outras proteínas. 26 Assim, a associação entre anemia e desnutrição pode não ser apenas devida a deficiências nutricionais, mas também a doenças crônicas associadas à desnutrição. 27 Além disso, a anemia ferropriva pode diminuir o número de linfócitos, explicando a associação independente entre anemia e desnutrição. 28

Notamos uma associação entre desnutrição e IMCs menores (“abaixo do peso” e “peso normal”), com maior risco em comparação a participantes obesos (IMC ≥ 30 kg/m ^2^ ). A prevalência de desnutrição no grupo abaixo do peso era esperada, visto que o IMC abaixo de 18,5 kg/m ^2^ frequentemente é um critério diagnóstico. No entanto, mesmo indivíduos com peso normal apresentaram maiores taxas de desnutrição laboratorial. Isso pode ser devido à inatividade relacionada à osteoartrite que leva a doenças crônicas, perda de massa magra e reposição por gordura, contribuindo para a desnutrição apesar do IMC normal. Em pacientes submetidos à artroplastia, indivíduos com peso normal apresentaram maior risco de desnutrição do que pacientes com sobrepeso e obesos (51% *versus* 27% e 32%, *p* = 0,0012 e 0,0023). 29 As alterações relacionadas à idade também apoiam o uso da classificação do IMC da OPAS em idosos, que identifica melhor os indivíduos abaixo do peso em risco nutricional e está associada à mortalidade maior. 30

Curiosamente, o diabetes parece ser um fator de proteção contra a desnutrição. Em pacientes submetidos à cirurgia da coluna lombar, o diabetes insulinodependente foi associado a taxas maiores de hipoalbuminemia, enquanto o diabetes não insulinodependente apresentou taxas semelhantes às dos não diabéticos. 31 Os estudos sobre diabetes e desnutrição têm resultados mistos. Um estudo austríaco com pacientes hospitalizados e geriátricos constatou que o diabetes é um fator de proteção segundo o escore MUST, aproximando-se da significância estatística (OR = 0,883, *p* = 0,06). 32 Por outro lado, o diabetes foi um fator de risco independente para a desnutrição em pacientes com carcinoma renal avaliados pela escala SGA. 33 A maioria dos estudos não encontrou associação significativa entre diabetes e risco de desnutrição. 34 35 Em nossa população, fatores como maior foco em proteínas alimentares e monitoramento nutricional mais rigoroso em pacientes diabéticos de alto risco podem ter contribuído para o melhor estado nutricional. Além disso, pode haver viés diagnóstico, já que indivíduos com *status* socioeconômico mais elevado podem ter melhor nutrição, maior acesso a cuidados de saúde e maior probabilidade de ter ou relatar o diagnóstico de diabetes.

A desnutrição e os níveis de marcadores séricos não foram fatores de risco independentes para complicações após a ATJ. As únicas associações observadas foram entre desnutrição e níveis de transferrina com o tempo de internação pós-operatória na análise bivariada, provavelmente devido a fatores de confusão como idade e anemia. Fu et al. 13 constataram que a hipoalbuminemia (albumina < 3,5 g/dL) estava associada ao tempo total de internação, mas apenas na análise bivariada. No entanto, a relação independente entre anemia e prevalência de desnutrição, juntamente com o aumento do tempo de internação pós-operatória, destaca que a anemia pré-operatória é um fator de risco modificável, porém frequentemente negligenciado. 14

Diversos estudos indicaram que a desnutrição aumenta o risco de complicações infecciosas e não infecciosas após a artroplastia. Em um estudo com 9.001 pacientes submetidos à artroplastia, aqueles com concentração de albumina < 3,5 g/dl apresentaram maior probabilidade de desenvolver IAP profunda (OR ajustada de 4,69, *p* < 0,001). 36 A taxa de infecção em pacientes com albumina baixa (7,3%) foi significativamente maior do que em nosso estudo (3,3%). Essa discrepância pode ser decorrente da inclusão de artroplastias de quadril, particularmente cirurgias urgentes em casos de fraturas proximais do fêmur, que apresentam maior risco de IAP precoce em comparação a procedimentos eletivos. 37

Bohl et al. 14 identificaram a concentração sérica de albumina como possível marcador de risco para IAP, complicações perioperatórias e readmissão. Estes autores notaram uma associação entre hipoalbuminemia, IMC extremo (< 18,5 kg/m ^2^ e > 40 kg/m ^2^ ) e idade avançada (> 70 anos). Esses resultados foram corroborados por Fu et al., 13 que relataram maiores taxas de complicações sépticas e assépticas em pacientes submetidos à ATJ. No entanto, Morey et al. 20 não observaram impacto da desnutrição nas taxas de infecção ou nos desfechos funcionais em 3.169 pacientes submetidos à ATJ, sugerindo que a avaliação nutricional por meio de parâmetros laboratoriais pode não estratificar o risco de infecção após a ATJ de maneira eficaz.

Uma revisão sistemática com meta-análise de 2019 avaliou 20 estudos sobre os efeitos da desnutrição definida laboratorialmente em pacientes submetidos à ATJ primária ou de revisão ou à artroplastia total de quadril (ATQ). Entre os 11 estudos com mais de 100.000 pacientes submetidos à ATJ primária, a prevalência combinada de desnutrição laboratorial foi de 11,6%. Apenas um estudo não observou associação entre desnutrição e complicações pós-operatórias. A meta-análise incluiu oito estudos comparáveis que revelaram uma associação significativa entre o nível de albumina < 3,5 g/dL e complicações da ferida operatória em pacientes submetidos à artroplastia (OR, 2,176, IC de 95%, 1,916-2,471). No entanto, a revisão não abordou o viés de publicação ou a heterogeneidade dos artigos. As limitações incluíram inconsistências nas definições de desnutrição, variações nos desfechos avaliados e dados escassos sobre o momento da coleta dos marcadores de desnutrição. 38

Este estudo apresenta diversas limitações. Obtivemos resultados analisáveis de albumina, transferrina e linfócitos de 68,9%, 55,2% e 95,7% da amostra, respectivamente. Estudos semelhantes relataram perdas de 30% a 70%, uma limitação comum neste delineamento experimental. 19 36 Apesar do tamanho significativo da amostra, apenas 18 casos de IAP precoce foram identificados, limitando a análise dos fatores de risco para infecção. A ausência de outras avaliações de desnutrição, como questionários nutricionais e análise da composição corporal, pode ter levado a um viés de classificação, com categorização errônea de pacientes desnutridos como eutróficos e vice-versa. No entanto, os pontos fortes incluem o foco na ATJ, o uso da classificação da OPAS para avaliação do risco nutricional pelo IMC em idosos e a restrição das datas dos exames laboratoriais em 6 meses antes do procedimento. Além disso, utilizamos os três parâmetros laboratoriais mais reconhecidos de desnutrição—albumina, transferrina e linfócitos—permitindo a avaliação da eficácia individual e combinada de cada critério.

## Conclusões

A prevalência de alterações no número de linfócitos e nas concentrações séricas de albumina e transferrina em pacientes submetidos à ATJ foi de 17,7%, com taxas maiores em pacientes mais idosos, anêmicos e com IMC normal ou baixo. A identificação desses grupos pode aprimorar o manejo pré-operatório por meio de intervenções nutricionais para correção parcial ou completa antes da cirurgia. Mais estudos com maior poder estatístico são necessários para avaliar a associação entre a desnutrição, independentemente da definição utilizada, e desfechos adversos após a ATJ.
