# Supplementary material for: Lymphocyte Count, Serum Albumin and Transferrin Levels in Patients Undergoing Total Knee Arthroplasty
Source: Rev Bras Ortop (Sao Paulo). 2025 Jul 25;60(2):s00451809530. [Article in Portuguese] doi: 10.1055/s-0045-1809530 (PMC12302332; doi:10.1055/s-0045-1809530)
Supplement: Supplementary file 1 — Supplementary Material [file 10-1055-s-0045-1809530_s2400329pt.pdf]

Tabela Suplementar S1 Estatística descritiva da amostra ($n = 2.080$)

	Frequência ou Mediana	% ou IIQ
Sexo		
Feminino	1.530	73,6%
Masculino	550	26,4%
Categoria de IMC OMS/OPAS ($n = 1.861$)		
Obesidade	1.025	55,1%
Sobrepeso	327	17,6%
Peso normal	435	23,4%
Subpeso	74	4,0%
ASA ($n = 1.522$)		
I	316	20,8%
II	1.194	78,4%
III	12	0,8%
Hipertensão arterial sistêmica ($n = 2.077$)		
Não	543	26,1%
Sim	1.534	73,9%
Diabetes mellitus		
Não	1.596	76,7%
Sim	484	23,3%
Cardiopatia		
Não	1.949	93,7%
Sim	131	6,3%
Dislipidemia		
Não	1.979	95,1%
Sim	101	4,9%
Artrite reumatoide ($n = 2.079$)		
Não	1.952	93,9%
Sim	127	6,1%
Anemia ($n = 1.797$)		
Não	1.389	77,3%
Sim	408	22,7%
Infecção periprostética precoce		
Não	2.037	97,9%
Sim	43	2,1%
Readmissão		
Não	1.892	91,0%
Sim	188	9,0%
Readmissão por complicação da ferida local		
Não	1.970	94,7%
Sim	110	5,3%
Idade (anos)	68	(63–74)
Tempo de hospitalização (dias)	5	(4–8)
Dias entre a admissão e a cirurgia	1	(1–3)
Dias entre a cirurgia e a alta	4	(3–5)

Tabela Suplementar S1 (*Continuação*)

	Frequência ou Mediana	% ou IIQ
IMC (kg/m^2)	30.6	(27,1–34,5)
Volume de sangue transfundido (mL)	309	(269–563)

Abreviaturas: ASA, Pontuação de risco *da American Society of Anesthesiologists*; IMC, índice de massa corporal; IIQ, intervalo interquartil; OMS, Organização Mundial de Saúde; OPAS, Organização Pan-Americana de Saúde.

Tabela Suplementar S2 Estatística descritiva de marcadores laboratoriais de desnutrição (*n* = 2.080)

	Frequência ou Mediana	% ou IIQ
Albumina <3,5 g/dL (*n* = 1.433)		
Não	1.403	(97,9%)
Sim	30	(2,1%)
Linfócitos <1.500 células/mm^3 (*n* = 1.988)		
Não	1.783	(89,7%)
Sim	205	(10,3%)
Transferrina <200 mg/dL (*n* = 1.149)		
Não	1.065	(92,7%)
Sim	84	(7,3%)
Dados laboratoriais completos		
Não	981	(47,2%)
Sim	1.099	(52,8%)
Um ou mais critérios de desnutrição (*n* = 1.099)		
Não	904	(82,3%)
Sim	195	(17,7%)
Dois ou mais critérios de desnutrição (*n* = 1.099)		
Não	1.082	(98,5%)
Sim	17	(1,5%)
Albumina (g/dL) (*n* = 1.433)	4,0	(3,8–4,2)
Linfócitos (células/mm^3) (*n* = 1.988)	2.280,0	(1.863,0–2.820,0)
Transferrina (mg/dL) (*n* = 1.149)	249.0	(225,0–278,0)

Abreviaturas: IIQ, intervalo interquartil.

Tabela Suplementar S3 Análise bivariada do tempo de internação

	Duração da internação após a ATJ primária (dias)	Valor de p
	Mediana (IIQ)	
Albumina <3,5 g/dL		
Sim	4 (3–6)	0,149
Não	3 (3–5)	
Transferrina <200 mg/dL		
Sim	4 (3–6)	**0,021**
Não	3 (3–5)	
Linfócitos <1.500 células/mm^3		
Sim	4 (3–5)	0,055
Não	3 (3–5)	

(*Continued*)

Tabela Suplementar S3 (*Continuação*)

	Duração da internação após a ATJ primária (dias)	Valor de p
	**Mediana (IIQ)**	
Sexo		
Feminino	4 (3–5)	0,336
Masculino	3.5 (3–5)	
ASA		
I ($n = 316$)	3 (2.5–4)	**0,002**
II ($n = 1.194$)	4 (3–5)	
III ($n = 12$)	5 (3.5–7)	
Idade		
<65 ($n = 689$)	3 (3–4)	$<$**0,001**
≥ 65 e <72 ($n = 673$)	4 (3–5)	
≤ 72 ($n = 718$)	4 (3–5)	
Transfusão de sangue		
Sim	6 (4–9)	$<$**0,001**
Não	3 (3–4)	
Infecção periprostética precoce		
Sim	4 (3–6)	0,078
Não	4 (3–5)	
Hipertensão arterial sistêmica		
Sim	4 (3–5)	0,253
Não	3 (3–5)	
Diabetes mellitus		
Sim	4 (3–5)	0,663
Não	3 (3–5)	
Cardiopatia		
Sim	4 (3–5)	**0,014**
Não	4 (3–5)	
Dor precordial		
Sim	3 (2–7)	0,617
Não	4 (3–5)	
Dislipidemia		
Sim	3 (3–5)	0,058
Não	4 (3–5)	
Acidente vascular cerebral		
Sim	5 (3–5)	0,127
Não	4 (3–5)	
Tumor		
Sim	3 (3–6)	0,591
Não	4 (3–5)	
Desnutrição		
Sim	4 (3–5)	**0,025**
Não	3 (3–5)	

Tabela Suplementar S3 (*Continuação*)

	Duração da internação após a ATJ primária (dias)	Valor de p
	**Mediana (IIQ)**	
IMC		
Subpeso	4 (3–5)	0,155
Peso normal	4 (3–5)	
Sobrepeso	3 (3–4)	
Obesidade	3 (3–5)	
Anemia		
Sim	4 (3–6)	<**0,001**
Não	3 (3–5)	

Abreviaturas: ATJ, Artroplastia total de joelho; ASA, pontuação de risco *da American Society of Anesthesiologists*; IIQ, intervalo interquartil, IMC, índice de massa corporal.
